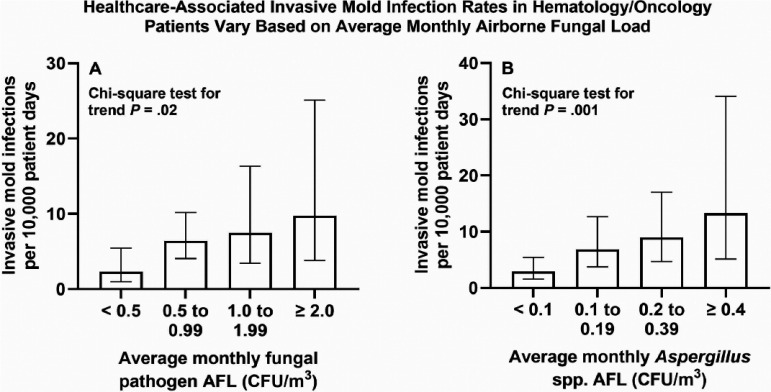# Environmental Surveillance for Airborne Mold Spores and Healthcare-Associated Invasive Mold Infections

**DOI:** 10.1017/ash.2025.224

**Published:** 2025-09-24

**Authors:** Bethany Phillips, Zachary Most, Bryan Connors, Pat Jackson, Michael Sebert

**Affiliations:** 1 Advocate Health; 2 University of Texas Southwestern Medical Center; 3 Environmental Health and Engineering; 4 Scottish Rite for Children; 5 UT Southwestern Medical Center

## Abstract

**Background:** The role of environmental sampling to monitor airborne fungal loads (AFL) in healthcare facilities is controversial due to a paucity of data to guide the interpretation of results. Systematic surveillance for infections that might result from exposure to airborne fungal spores is furthermore limited by the lack of standardized definitions for healthcare-associated invasive mold infections (IMI).

Setting: 490-bed tertiary-care pediatric hospital **Methods:** Retrospective ecological study of the relationship between AFL and IMI. Volumetric samples for fungal culture from 1000 L of air were obtained approximately monthly from November 2018 through October 2023 with a bioaerosol impactor on units caring for patients at risk for IMI. Fungi in environmental cultures were classified as opportunistic fungal pathogens versus those that are rarely pathogenic. Prospective surveillance was conducted using standard definitions for proven and probable IMI. Cases with symptom onset after one week of hospitalization or in patients with either a previous hospitalization or more than one ambulatory encounter at our facility during the month prior to admission were considered healthcare-associated events. Poisson regression was used to examine the association between AFL and monthly average IMI rates. AFL values were also categorized to analyze the association with IMI rates using the chi-square test for trend. **Results:** During the period of AFL surveillance, 51 healthcare-associated proven or probable IMI were identified of which 33 were in hematology/oncology patients (including stem-cell transplant recipients) and 7 were in cardiac patients. The median total AFL on occupied inpatient units was 2 CFU/m3, and the most frequent pathogens identified were Penicillium species, dematiaceous molds, and Aspergillus species. No significant association was found between IMI rates and the average house-wide AFL for opportunistic fungal pathogens. The hematology/oncology IMI rate, however, increased by 1.48-fold (95% CI 1.00-2.19, P = .05) in association with an increase of 1 CFU/m3 in the pathogen AFL on units caring for these patients. The local AFL of Aspergillus species demonstrated an even stronger association with the hematology/oncology IMI rate (15.9-fold increase for an increase of 1 CFU/m3 [95% CI 2.8-90.7, P = .002]). The figure summarizes trends in hematology/oncology IMI rates across different ranges of average monthly AFL values. **Conclusions:** Environmental surveillance for AFL on appropriate hospital units may identify periods of increased risk for IMI among hematology/oncology patients. Additional work is needed to define the role that routine AFL surveillance may serve in infection prevention activities for immunocompromised patients.

**Figure:**